# The Biomechanics of Amnion Rupture: An X-Ray Diffraction Study

**DOI:** 10.1371/journal.pone.0001147

**Published:** 2007-11-07

**Authors:** Che J. Connon, Takahiro Nakamura, Andy Hopkinson, Andrew Quantock, Naoto Yagi, James Doutch, Keith M. Meek

**Affiliations:** 1 School of Pharmacy, University of Reading, Reading, United Kingdom; 2 Department of Ophthalmology, Kyoto Prefectural University of Medicine, Kyoto, Japan; 3 Department of Ophthalmology, Nottingham University, Nottingham, United Kingdom; 4 School of Optometry and Vision Sciences, Cardiff University, Cardiff, United Kingdom; 5 Japan Synchrotron Radiation Research Institute, Hyogo, Japan; Illinois Institute of Technology, United States of America

## Abstract

Pre-term birth is the leading cause of perinatal and neonatal mortality, 40% of which are attributed to the pre-term premature rupture of amnion. Rupture of amnion is thought to be associated with a corresponding decrease in the extracellular collagen content and/or increase in collagenase activity. However, there is very little information concerning the detailed organisation of fibrillar collagen in amnion and how this might influence rupture. Here we identify a loss of lattice like arrangement in collagen organisation from areas near to the rupture site, and present a 9% increase in fibril spacing and a 50% decrease in fibrillar organisation using quantitative measurements gained by transmission electron microscopy and the novel application of synchrotron X-ray diffraction. These data provide an accurate insight into the biomechanical process of amnion rupture and highlight X-ray diffraction as a new and powerful tool in our understanding of this process.

## Introduction

The extraplacental fetal membranes which surround the amniotic cavity are composed of the amnion and the chorion [1]. These membranes form an adjustable container for a developing and moving fetus. They are genetically identical to the fetus, but have a limited lifetime, existing only to the point of programmed rupture at term, which is a normal event during the first stage of labour. In contrast, preterm premature rupture precedes 30–40 per cent of preterm births and carries significant risks for the infant [2], [3]. Therefore, an understanding of the basic structural components of the human fetal membranes, and how they adapt to changing needs as the uterine contents enlarge, is central to the eventual control of this major health problem.

The most structurally robust of the fetal membranes is the amnion [4]. The amnion consists of a single layer of epithelial cells on a thick basement membrane which lies upon layers of collagenous tissue interspersed with mesenchymal cells. The compact and fibroblast layers of connective tissue beneath the basement membrane form the main fibrous skeleton of the amnion. Interstitial collagens (types I and III) predominate and form parallel bundles of collagen fibrils that maintain the mechanical integrity of the amnion [5].

It has been suggested that the rupture of the membranes is not solely a passive process related to myometrial contractions and cervical dilatation but that an enzymatic break-down of the membranes causes a local or global weakening of the tissue before rupture [6]. Although biochemical and microscopic perspectives have provided important insights into how the extracellular matrix of the amniotic membrane remodels during fetal development [6]–[11], these approaches have not yet explained how gross changes to the morphological and physical properties of the amnion, which must occur during the rupture process, result in a large defect in what had previously been intact tissue.

Non-crystalline X-ray diffraction (XRD) has been previously used to great effect in studying fibrillar collagen organisation in relation to development and disease within structural tissues such as the cornea, breast, bone and cartilage [12]–[15]. However, to the authors' knowledge there have been no reported XRD studies on the amniotic membrane. Low-angle XRD is a non-invasive technique which produces structural information on the average collagen fibril diameter, average spacing between fibrils and their arrangement within the tissue. Previous studies using electron microscopy do not apply directly to normal amniotic membrane because the specimen preparation procedures involve fixation, dehydration and chemical staining. XRD studies are important in this context because they can investigate normal intact structure at physiological hydration.

The aim of this study was to investigate the regular orientation of collagen fibrils within human amnion by XRD, complemented by transmission electron microscopy (TEM), to accurately quantify gross differences in collagen fibril organisation between areas near to and distant from the placenta at term.

## Materials and Methods

### Collection of Amniotic Membrane

Following elective Caesarean section at term, unlinked anonymised samples of amniotic membrane from nine patients' placentas, were collected from the Department of Obstetrics and Gynaecology, Queens Medical Centre, Nottingham, UK with approval from the Local Research Ethics Committee (Nottingham). Within local operational and ethical guidelines it is acceptable to use human amniotic membrane (material surplus to clinical requirements) for research without consent if it is anonymous and unlinked.

The chorion was separated manually from the amnion and discarded. Under sterile conditions samples (4 cm×4 cm) of amnion were taken from areas proximal to the placental disc (proximal amnion) and approximately 10 cm distal to the placental disc (distal amnion). The samples were chosen from areas of the amnion which were coherent, translucent and without blood clots. The dissected amniotic samples were washed with phosphate-buffered saline (PBS) containing antibiotics (5 ml of 0.5% levofloxacin) to remove cellular debris and blood and stored at −80°C in Dulbecco's modified Eagle's medium (GibcoBRL, Rockville, MD) and glycerol (Wako Pure Chemical Industries, Osaka, Japan) in the ratio of 1∶1 by volume.

### Transmission Electron Microscopy (TEM)

A 1 cm^2^ sample of each proximal and distal amniotic tissue was thawed, and then fixed in 2% glutaraldehyde, 0.1M PBS for 1 hour. The tissues were then rinsed in PBS then treated with 1% osmium tetroxide for 1 hour, followed by dehydration in ethanol. The tissues were then cut into 1 mm×3 mm strips, embedded in Araldite (Agar Scientific, UK) and ultrathin sections cut at 90 nm. The sections, held on uncoated grids, were then counterstained with uranyl acetate for 15 min at 40°C [16] and lead citrate for 5 min [17]. Collagen fibrils within the fibroblast layer in both proximal and distal AM were studied using a Philips EM208 transmission electron microscope.

Quantitative image analysis was achieved by digitizing the micrographs at 600dpi using a conventional flatbed scanner connected to a personal computer equipped with the image analysis software, Image J (National Institutes of Health). Once digitized, the background noise within each image was reduced while retaining the size and position of each fibril [18]. These images were then binarized and their relative fibril positions calculated. Each micrograph analyzed contained approximately 300 fibrils in cross section. A representative measurement of the relative position of collagen fibrils in cross section was determined from the proximal and distal amniotic tissue. This information was then used to calculate a radial distribution function, *g(r)*, a mathematical description of the positions of the fibrils with respect to one another.

### Calculation of Radial Distribution Function

The radial distribution, *g(r)*, is a statistical measurement of the average number density of fibril centers at a given distance, *r*, from any other fibril center, relative to the bulk fibril number density ρ (the number of fibrils per unit area in cross-section), resulting in a histogram of *g(r)* plotted against an increasing *r* [19]. ρ was calculated from the fibril positions taken from two representative TEM digital images of fibrils in cross section. A radial distribution pattern with a distinct peak followed by smaller undulations and eventual stability indicates that neighboring fibrils are relatively uniformly spaced, but without any long-range order. A flatter peak and lower bulk fibril number density order reflects a lessening in short-range order within a structure.

### Synchrotron X-ray Diffraction

Low-angle X-ray diffraction was carried out at SRS, Daresbury, UK and SPring-8, Hyogo Prefecture, Japan. The unfixed proximal (n = 9) and distal (n = 9) amniotic tissues were defrosted, washed in PBS then folded in half four times (each successive axis of symmetry was perpendicular to the last) before being mounted between Mylar windows in clear plastic cells to avoid tissue dehydration during exposure to the X-ray beam. The plastic cells were placed in the X-ray beam so that the X-rays passed through the center of the folded tissue. Low-angle patterns were collected at beam-line stations 2.1 (SRS) and 40xu (SPring 8) with finely focused X-ray beams of λ = 1.543Å, 1 mm×3 mm and λ = 1.181Å, 25 µm×25 µm, exposure times of 180 and 1 second(s), at distances of 6.25 m and 2 m respectively. Diffraction patterns were recorded on 2D image plates 8 m and 2 m behind the specimen respectively. Rat-tail tendon was used to calibrate data [12]. The interfibrillar Bragg spacing was calculated from the position of the innermost equatorial reflection after background subtraction (using a similarly obtained background pattern from the Mylar windows of the empty specimen holder) and division of fibril transform [20], [21].

Wet weights of the samples were taken before and after exposure to the X–ray beam and averaged. Hydration (H) values were calculated by H = (wet weight-dry weight)/dry weight.

## Results

### Transmission electron microscopy

**Figure 1:**
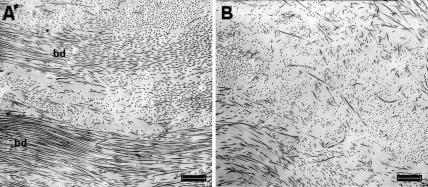
Transmission electron micrographs from the fibroblastic layer of amnion. Collagen fibrils appear more regularly aligned, closer together and in more obvious bundles (bd) within the stroma of amnion proximal to the placenta (a), than those shown in the tissue distal to the placenta (b). Scale bar = 1 µm.

**Figure 2:**
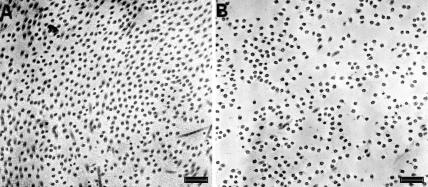
High magnification transmission electron micrographs of amnion collagen fibrils in cross section. No difference in fibril diameter was observed between samples proximal (a) and distal (b) to the placenta. However a marked decrease in fibril packing can be seen in the amnion distal to the placenta. Scale bar = 300 nm.

Electron micrographs from the fibroblast layer (mid stroma) of amnion proximal and distal to the rupture site displayed collagen fibrils arranged loosely into bundles, each bundle containing fibrils with a similar axial orientation. Between adjoining bundles (and the similarly orientated fibrils within) there appeared to be large angle difference. However, the fibrils within distal amnion appeared to lack qualitatively the same degree of organisation because the bundles were less apparent and the spacing between neighbouring fibrils seemed increased (Fig. 1). High magnification images of intra-bundle collagen fibrils in cross section from both proximal and distal amnion were compared and no difference in fibril diameter observed (Fig. 2).

### Radial distribution function (RDF)

**Figure 3:**
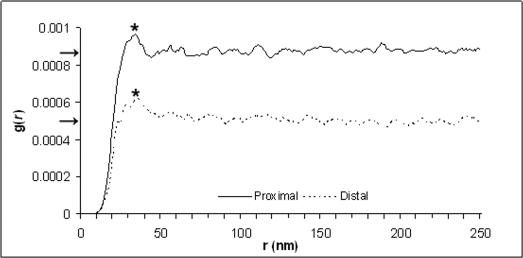
Radial distribution functions (RDF) calculated from positions of amnion fibrils in cross section from representative images. The primary RDF peak (*) from amnion distal to the placenta was lower and broader than the corresponding peak from amnion proximal to the placenta indicating less regular fibril spacing. The bulk fibril number density (ρ) was also decreased in distal amniotic tissue (arrows).

The RDF shows a distinct peak in both tissue types as the value of g(*r*) rises from zero (for r = 0) upwards until a nearest neighbour distance is reached (proximal amnion = 34 nm, distal amnion = 37 nm). The primary peak is followed by smaller undulations (2^nd^ and 3^rd^ peaks) before stabilising to a constant value, the bulk fibril number density, in the radial distribution histogram (Fig. 3). The difference in primary peak size and shape reflects upon the degree of fibrillar order within each tissue. The flatter peak shown in the distal AM indicates a less well ordered internal structure. The loss of fibril compaction seen by TEM in distal amnion when compared to proximal amnion (Fig. 2) was also quantified by the radial distribution function as the bulk fibril number density (number of fibrils per unit area) and was shown to have decreased by 45% (proximal amnion = 867×10^−6^, distal amnion = 480×10^−6^)

### X-ray diffraction (XRD)

**Figure 4:**
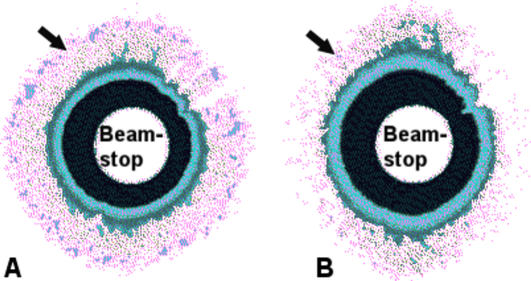
Synchrotron X-ray diffraction patterns from amnion. Representative (false colour) patterns collected from amnion proximal (a) and distal (b) to the placenta. Arrows indicate position of the interfibrillar reflection (magenta) other coloured rings close to the beamstop are background/backscatter. X-ray diffraction patterns displayed an anisotropic arrangement of collagen fibrils within the distal amniotic tissue.

**Figure 5:**
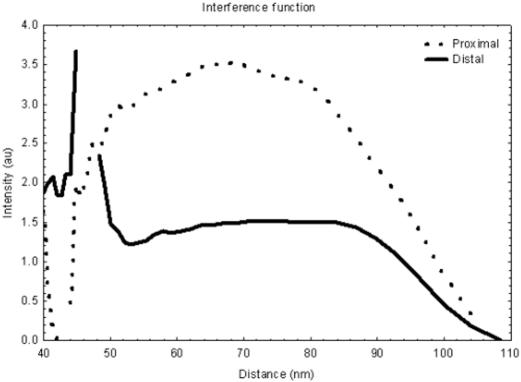
Intensity profiles across representative X-ray diffraction patterns collected from the amnion. Examination of intensity versus distance plots provided interfibrillar Bragg spacing measurements from amnion proximal (a) and distal (b) to the placenta. The lower broader interference function with a mean peak position of 75 nm from the distal amnion suggests that this region contains a less ordered fibril collagen arrangement and a higher mean interfibrillar Bragg spacing than that shown by proximal amniotic tissue.

The moderate lattice structure observed by TEM suggested that XRD patterns could be obtained from amnion. The subsequently collected XRD patterns display an equatorial reflection which corresponds to an interfibrillar Bragg spacing, an average measure of the lateral separation of neighbouring collagen fibrils (Fig. 4). The presence of a diffuse equatorial reflection in both tissue types corresponds to regular, periodic configuration of fibrils throughout the tissue volume investigated, indicating that a significant number of fibrils are aligned parallel to one another forming a loose arrangement with some degree of spatial order. Furthermore, the equatorial reflection from the distal amnion shows a pronounced anisotropic intensity, indicating that there are proportional more aligned fibrils running in one direction within the folded tissue (Fig. 4a). This anisotropic arrangement is less obvious in the proximal amnion (Fig. 4b). Line scans across the diffraction patterns reveal a similarity in the position and shape of the fibril transform (the amplitude of x-rays diffracted by a single fibril) suggesting no significant change in fibril diameter between the two areas of amnion similar to the results gained by TEM (Fig. 5). However, the line scans do show a dramatic difference in interfibrillar Bragg spacing between proximal and distal amnion. The interference function from the proximal tissue has a peak position of approximately 68 nm, whereas the distal amnion has a peak corresponding to an interfibrillar Bragg spacing of approximately 74 nm. Furthermore, quantification of the peaks shapes (height/width at half height) (proximal = 0.8, distal = 0.4) suggests that there are considerably less regularly spaced fibrils within the amnion distal to the placenta. Hydration values for the proximal and distal tissues were 5.3±0.6 and 4.3±0.5 respectively.

## Discussion

Our electron microscopy results and RDF calculations have allowed us to quantify the change in collagen organisation between amnion proximal and distal to the placenta and our results support the observations of Bou-Resli et al. [22] that within the fibroblast layer there are fewer collagenous fibers (per unit area) and less organization near the rupture site (distal amnion). However, we have also shown that this change in organisation is not influenced by fibril diameters as these remain unchanged, evidenced by our TEM and XRD results.

The presence of a second, and possibly third, RDF peak particularly from within the more organized proximal amnion tissue indicates that the fibrillar order may extend up to 100 nm from each fibril centre. Therefore, it seems probable that within the bundles of collagen fibrils there exists a loose lattice structure. The fact that we were able to obtain a first order equatorial X-ray reflection from amnion signifies that the packing of collagen fibrils within this tissue is indeed fairly regular.

The observation that the interfibrillar reflection from distal amnion is less intense than that from the proximal amnion tells us that either 1) the tissue is edematous at this point and collagen fibrils are less likely to exist in bundles, 2) collagen fibrils exist in groups and are regularly spaced, but not enough fibrils (scatterers) are present to give rise to a similarly intense first-order interfibrillar reflection, or 3) collagen fibrils do exist in bundles but within these groups the interfibrillar spacing is not particularly uniform. The first and second considerations are unlikely since both the proximal and distal tissues had similar hydration levels consistent with the findings of Halaburt et al. [23] and despite tissue near the rupture site being normally thinner than tissue near the placental margin [22], [23] the total amount of collagen per unit dry weight remains the same [23]. Therefore, the third explanation seems the most likely as this is supported by the increased average Bragg spacing, and the flatter RDF peaks.

Interestingly, the XRD results also uncovered a difference in collagen orientation between the two areas of amnion, whereby the distal amnion demonstrated a preferential orientation of its constituent collagen fibrils. It is tempting to speculate that this orientation is related to the biomechanics of amnion rupture as these fibrils are strongest axially, and directions of preferred fibril orientation thus associate with directions of heightened tissue strength implying that rupture might occur perpendicular to the fibril axis. However it is important to bear in mind that this orientation phenomenon may be an artifact of tissue thickness resulting from a change in preferential alignment of the collagen fibrils as a function of depth through the amniotic membrane or folding prior to XRD exposure as the initial fold was random with respect to orientation within the intact tissue.

Fibril orientation may play a role in reinforcing amnion structure but fibril diameters are also biomechanically important, because they determine the fibrils critical length [24], (*l*_c_) given by
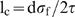
(1)where *d* is the fibril diameter, σ*_f_* is the fibril's tensile strength and τ is the shear stress exerted on the fibril by the ground substance (we define ground substance as being matrix elements other than fibrillar collagen). The critical length is the minimum fibril length required for effective tissue reinforcement [24]. As long as this condition is met, the tensile strength of the tissue (σ_t_) is determined by the volume fraction of collagen present (ß)
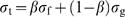
(2)where σ_f_ and σ_g_ are the tensile strengths of the fibrils and ground substance, respectively [24], [25]. Therefore, it is our hypothesis that a higher collagen fibril number density is necessary to maintain tissue strength, and a lower fibril density will subsequently contribute to the tissues rupture. Therefore, it is our hypothesis that the higher bulk fibril number density of collagen fibrils we have observed in the proximal amnion is necessary to maintain tissue strength. Thus, a lower number density would contribute to the membranes rupture. Inspection of equation 2 reveals that, for σ_f_>σ_g_, decreasing the volume fraction of collagen produces a proportional decrease in the mechanical strength of the tissue. Hence, we expect increased fibril spacing and thus decreased collagen volume fraction to result in a weaker amniotic membrane tissue distal to the placenta. Such a mechanism could help in our understanding of the causes of preterm rupture.

In conclusion this article provides proof of a previously unreported loose lattice structure exiting within amnion evidenced by electron microscopy and XRD, furthermore this lattice is diminished within tissue distal to the placenta. The loss of this regular structure may contribute to a weakening of the amnion facilitating its eventual rupture. Therefore XRD represents an untapped and powerful tool in our understanding of amnion structure and preterm rupture.
